# Long pentraxin PTX3 is upregulated systemically and centrally after experimental neurotrauma, but its depletion leaves unaltered sensorimotor deficits or histopathology

**DOI:** 10.1038/s41598-021-89032-7

**Published:** 2021-05-05

**Authors:** Marco Oggioni, Domenico Mercurio, Denise Minuta, Stefano Fumagalli, Katarzyna Popiolek-Barczyk, Marina Sironi, Agata Ciechanowska, Stefania Ippati, Daiana De Blasio, Carlo Perego, Joanna Mika, Cecilia Garlanda, Maria-Grazia De Simoni

**Affiliations:** 1 Istituto Di Ricerche Farmacologiche Mario Negri IRCCS, Via Mario Negri 2, 20156 Milan, Italy; 2 Humanitas Clinical and Research Center – IRCCS, via Manzoni 56, Rozzano - Milan, 20089 Italy; 3 Department of Pain Pharmacology, Maj Institute of Pharmacology Polish Academy of Sciences, Krakow, Poland; 4 Humanitas University, Department of Biomedical Sciences, Via Rita Levi Montalcini 4, Pieve Emanuele – Milan, 20090 Italy; 5 Present Address: San Raffaele Telethon Institute for Gene Therapy (SR-Tiget), San Raffaele Hospital, 20132 Milan, Italy; 6 Present Address: San Raffaele Scientific Institute, San Raffaele Hospital, 20132 Milan, Italy

**Keywords:** Neuroimmunology, Acute inflammation, Chronic inflammation

## Abstract

Long pentraxin PTX3, a pattern recognition molecule involved in innate immune responses, is upregulated by pro-inflammatory stimuli, contributors to secondary damage in traumatic brain injury (TBI). We analyzed PTX3 involvement in mice subjected to controlled cortical impact, a clinically relevant TBI mouse model. We measured PTX3 mRNA and protein in the brain and its circulating levels at different time point post-injury, and assessed behavioral deficits and brain damage progression in PTX3 KO mice. PTX3 circulating levels significantly increased 1–3 weeks after injury. In the brain, *PTX3* mRNA was upregulated in different brain areas starting from 24 h and up to 5 weeks post-injury. PTX3 protein significantly increased in the brain cortex up to 3 weeks post-injury. Immunohistochemical analysis showed that, 48 h after TBI, PTX3 was localized in proximity of neutrophils, likely on neutrophils extracellular traps (NETs), while 1- and 2- weeks post-injury PTX3 co-localized with fibrin deposits. Genetic depletion of PTX3 did not affect sensorimotor deficits up to 5 weeks post-injury. At this time-point lesion volume and neuronal count, axonal damage, collagen deposition, astrogliosis, microglia activation and phagocytosis were not different in KO compared to WT mice. Members of the long pentraxin family, neuronal pentraxin 1 (nPTX1) and pentraxin 4 (PTX4) were also over-expressed in the traumatized brain, but not neuronal pentraxin 2 (nPTX2) or short pentraxins C-reactive protein (CRP) and serum amyloid P-component (SAP). The long-lasting pattern of activation of PTX3 in brain and blood supports its specific involvement in TBI. The lack of a clear-cut phenotype in PTX3 KO mice may depend on the different roles of this protein, possibly involved in inflammation early after injury and in repair processes later on, suggesting distinct functions in acute phases versus sub-acute or chronic phases. Brain long pentraxins, such as PTX4—shown here to be overexpressed in the brain after TBI—may compensate for PTX3 absence.

## Introduction

Traumatic brain injury (TBI) is the leading cause of mortality in young adults and a major cause of death and disability across all ages in all countries, with no specific agent proved to be effective in dampening the neurologic *sequelae* post-injury^1^. Inflammatory processes contribute to outcome following TBI. Modulation of these processes represents a possibility to limit secondary neuronal injury and to improve patient outcome^2^ although a detailed knowledge of the processes required to identify therapeutical targets is still largely lacking.

Pentraxins are a superfamily of phylogenetically conserved humoral mediators of innate immunity^3^ that share conserved sequences. They include short (such as C-reactive protein, CRP, serum amyloid P-component, SAP) and long (such as pentraxin 3 PTX3, neuronal pentraxin 1, nPTX1, neuronal pentraxin 2, nPTX2 and pentraxin 4, PTX4) members. Among long pentraxins, PTX3 is an essential mediator of innate resistance to selected pathogens of fungal, bacterial and viral origin, and is involved in regulation of inflammation, tissue remodeling and cancer^4^. PTX3 acts as fine tuner of inflammation in several diseases, with detrimental or beneficial effects that depend on the nature of the insult^4–6^. PTX3 regulatory role for inflammatory responses may be played in a time-dependent manner, i.e. limiting neutrophil recruitment at acute phases and orchestrating tissue repair processes at chronic phases^7–9^. In brain disorders, available evidence indicates that PTX3 may play a protective role preserving neuron survival after seizures or stroke^10,11^. PTX3 was also proposed to dampen neutrophil recruitment to injury site after cerebral ischemia, maintain blood–brain barrier integrity, regulate glial scar formation and promote injury resolution thus highlighting its involvement in brain repair and recovery^12–14^. PTX3 is also know to modulate inflammation mediated by the complement system^15,16^, a key contributor to TBI pathogenesis in experimental models^17–19^ and in TBI patients^20–22^.

The potential involvement of PTX3 in TBI is directly suggested by the observation that serum levels of PTX3 increase significantly after severe TBI in patients and are independently associated with hospital mortality^23^. However, the role of PTX3 in TBI remains poorly understood^24,25^. We therefore investigated PTX3 in mice subjected to controlled cortical impact (CCI), a clinically relevant model of TBI^26–29^. We analyzed PTX3 plasma and brain presence in acute (as early as 30 min), sub-acute (1–3 weeks) and chronic (4–5 weeks) phases after TBI. We identified PTX3 localization in association with different cell populations present in the injured brain, namely neutrophils, astrocytes, neurons, microglia and endothelial cells, as well as with fibrin(ogen) deposits. Then, to further investigate PTX3 relevance in TBI pathophysiology, we compared damage progression, assessed by sensorimotor and histopathological analysis in wild-type (WT) and PTX3 depleted (PTX3 KO) mice. Our data reports a long-lasting pattern of activation of PTX3 in brain and blood following TBI, and supports the hypothesis that PTX3 contributes to the progression of the lesion with effects varying over time, enhancing inflammation early after injury and fostering repair processes later on.

## Methods

### Mice

Procedures involving animals and their care were conducted in conformity with institutional guidelines in compliance with national and international laws and policies. The experimental protocols were approved by Ethical Committee at the Istituto di Ricerche Farmacologiche Mario Negri IRCCS and by Italian Ministry of Health (prot. 9F5F5.81 authorization nº 753/2017-PR). We used male 9 week old C57BL/6 J mice (Charles Rivers-Italy) weighting 22–28 g, either WT or with targeted depletion of PTX3 (PTX3 KO, C57BL/6 J inbred genetic background, Humanitas Clinical and Research Center)^30^. The protocols and details of this report are in accordance with ARRIVE guidelines (http://www.nc3rs.org.uk/page.asp?id=1357, check list provided as Supplementary file).

### Experimental TBI

Mice were anesthetized with isoflurane inhalation (induction 5%; maintenance 2%) in an N_2_O/O_2_ (70%/30%) mixture and placed in a stereotactic frame. Mice were then subjected to craniotomy followed by induction of CCI brain injury as previously described^26,26,27,29^. Briefly, the injury was induced using a 3 mm diameter rigid impactor driven by a pneumatic piston rigidly mounted at an angle of 20° from the vertical plane and applied vertically to the exposed dura mater, between bregma and lambda, over the left pareto-temporal cortex. We set an impactor velocity of 5 m/s and deformation depth 1 mm, resulting in a severe level of injury^18,31^. The craniotomy was then covered with a cranioplasty and the scalp sutured. Sham-operated mice received identical anaesthesia and surgery without craniotomy and brain injury.

### Tissue processing

#### Collection and processing of brain areas

For gene expression studies, selected brain areas were collected from sham and TBI mice sacrificed at the following time points: 24 h (h), 96 h, 1 week (w), 2w and 5w. Tissues from the ipsilateral cortex (including all the tissue above the rhinal fissure), striatum, hippocampus and from the thalamus were dissected out, rapidly loaded into RNA-later (Ambion, Inc, Austin, USA), frozen, shipped at Department of Pain Pharmacology (Krakow, Poland) and stored at − 80 °C until use.

#### Collection and processing of blood samples

Blood was obtained from the vena cava of anesthetized mice. Clotting and complement activation were prevented by collecting samples in 10 mM ethylenediaminetetraacetic acid (EDTA) and 0.125% polybrene (Sigma-Aldrich, St. Louis, MO). Plasma was centrifuged at 2000 × g for 15 min at 4 °C and supernatant divided into aliquots and stored at − 80 °C before analysis^32^.

#### Collection and processing of brain by perfusion/fixation

At 30 min (’), 24 h, 48 h, 96 h, 1w, 2w, 3w, 4w, 5w after surgery, under deep anaesthesia (Ketamine 20 mg + Medetomidine 0.2 mg), animals were transcardially perfused with 30 mL of phosphate buffer saline (PBS) 0.1 mol/L, pH 7.4, followed by 60 mL of chilled paraformaldehyde (4%) in PBS. The brains were carefully removed from the skull and post-fixed for 6 h at 4 °C, then transferred to 30% sucrose in 0.1 mol/L phosphate buffer for 24 h until equilibration. The brains were frozen by immersion in isopentane at − 45 °C for 3 min before being sealed into vials and stored at − 80 °C until use. Coronal brain 20 μm-thick cryosections were cut serially (from bregma + 1.2 mm to bregma − 4 mm) at 200 µm intervals for histological analysis^33^.

### Analysis of pentraxin family gene expression by RT-qPCR

For RT-qPCR, total RNA was extracted according to Chomczynski & Sacchi (1987)^34^ with TRIzol reagent (Invitrogen, Carlsbad, USA) as previously described^35^. The RNA concentration was measured using a DeNovix DS-11 Spectrophotometer (DeNovix Inc., Wilmington, USA). Reverse transcription was performed on 1000 ng of total RNA using Omniscript Reverse Transcriptase (Qiagen Inc., Hilden, Germany) at 37 °C for 60 min. The resulting cDNA was diluted 1:10 with H_2_O. RT-qPCR was performed using Assay-On-Demand TaqMan probes according to the manufacturer's protocol (Applied Biosystems, Foster City, USA) and was run on an iCycler device (BioRad, Hercules, Warsaw, Poland). The following TaqMan primers were used: Mm00446968_m1 (*Hprt*), Mm00476505_m1 (*nPtx1*), Mm00479438_m1 (*nPtx2*), Mm00477268_m1 (*Ptx3*), Mm03990600_m1 (*Ptx4*), Mm00488099_g1 (*SAP*), Mm00432680_g1 (*CRP*). The expression of the *Hprt* transcript (a housekeeping gene) was quantified to control for variations in the amount of cDNA. The cycle threshold values were calculated automatically using iCycler IQ 3.0 software with the default parameters. The abundance of RNA was calculated as 2^−(threshold cycle)^.

### Enzyme-linked immunosorbent assay (ELISA)

Murine PTX3 was measured by ELISA (DuoSet ELISA Development System, R&D Systems, Minneapolis, MN, USA) according to manufacturer’s instruction) at 30′, 24 h, 48 h, 96 h, 1w, 2w, 3w and 5w after TBI. The ELISA assay did not cross react with short pentraxins CRP and SAP^36^.

### Quantification of PTX3 presence in the brain cortex

The brain coronal Sects. (20 µm) were incubated overnight at 4 °C with primary polyclonal goat antibody anti-mouse PTX3 (0.2 mg/mL, 1:100; R&D Systems, Minneapolis, MN, USA) followed by a secondary biotinylated antibody against goat or rat IgG. Positive cells were stained with Tyramide Fluorescein (1:300, Perkin Elmer, Milan, Italy). Cell nuclei were stained with 40–6-diamidino-2-phenylindole (Hoechst, 1 mg/ml, Invitrogen, Carlsbad, CA). For negative control staining, the primary antibody was omitted, and no staining was observed. Three 20 μm-thick coronal sections at 0.4, 1.6, and 2.8 mm posterior to bregma were selected from each mouse brain for quantification. Confocal microscopy was done with a Nikon A1 confocal scan unit with a 20 × 0.5 numerical aperture (NA) objective, managed by NIS elements software. Tissues were imaged at laser excitation of 405 nm (for nuclei) and 488 nm (for PTX3)^37^. Image acquisition was done at 12-bit, keeping the fluorescent signal in a non-saturated range (0–1000 greyscale values). The acquisition was done over an area sized 2 × 2.5 mm, positioned in the ipsilateral hemisphere along the cortical region proximal to the lesion, with a pixel size of 0.62 µm. Acquisition was done over 8.3 µm thick stacks, with a step size of 2.075 µm. The different focal planes were merged into a single stack by maximum intensity projection to ensure consistent focus throughout the sample. Immunostaining for PTX3 was quantified by assessing fluorescence intensity using Fiji software. To subtract the background signal, a minimum threshold was applied based in the highest grayscale value of background^18^. PTX3 signal was reported as integrated density (ID).

### Immunofluorescence and confocal analysis

Immunofluorescence was performed on 20 µm coronal sections. After thorough washings with PBS 0.01 M, sections were incubated with blocking solution with 10% fetal horse serum (FHS) and Triton X-100 0.2% for 1 h at RT and then with goat anti-mouse PTX3 (0.2 mg/mL, 1:100; R&D Systems, Minneapolis, MN, USA). Positive cells were stained with Tyramide Fluorescein or Cyanine 5 (1:300, Perkin Elmer, Milan, Italy). The following primary antibodies were incubated for 1 h: rabbit anti-neutrophil Elastase (1:300; Abcam, Cambridge, UK) in Low Cross Buffer (CANDOR Bioscience, Wangen, Germany), mouse anti-mouse NeuN (10 μg/mL, 1:250; Merck Millipore; Burlington; MA; USA), rat anti-mouse CD11b (1 μg/mL; 1:200; Abcam, Cambridge, UK), goat anti-mouse glial fibrillary acid protein (GFAP, 0.5 μg/mL, 1:2000; Chemicon), rat anti-mouse CD31 (15.625 µg/ml, 1:100; BD Bioscience, San Jose, CA, USA), rabbit anti-human Fibrinogen FITC (which recognizes both native fibrinogen and its fragments; 100 mg/L, 1:50; Dako, Santa Clara, CA, USA). Primary antibodies were followed by appropriate Alexa 488-, Alexa-546 or Alexa 555-conjugated secondary antibodies raised in goat (4 µg/mL, Life Sciences, Hercules, CA, USA). Cell nuclei were stained with Hoechst (1 mg/ml, Invitrogen, Carlsbad, CA, USA). For negative control staining, the primary antibodies were omitted, and no staining was observed. Immunofluorescence was acquired using a scanning sequential mode to avoid bleed-through effects by an IX81 microscope equipped with a confocal scan unit FV500 with 4 laser lines: Ar-Kr (488 nm), He–Ne red (646 nm), and He–Ne green (532 nm) (Olympus, Tokyo, Japan) and a UV diode. Three-dimensional volumes were acquired over 10 µm stacks, with 0.23 µm step size. Images were managed and elaborated to obtain three-dimensional renderings with Imaris v.6 (Bitplane) and GNU Image Manipulation Program (GIMP).

### Sensorimotor deficits

#### Composite neuroscore

Mice were scored from 4 (normal) to 0 (severely impaired) for each of the following: (1) forelimb function during walking on the grid and flexion response during suspension by the tail; (2) hindlimb function during walking on the grid and extension during suspension by the tail;(3) resistance to lateral right and left push. The best total score is 12^18^.

#### Beam walk

The beam walk test measures the number of foot faults of the mouse walking twice on an elevated, narrow wooden beam (5 mm wide and 100 cm long). Before each test, mice are trained in three habituation trials. Data are expressed as the sum of the number of foot faults during the two tests. The best score is 0^18^.

### Histological stainings

Cryostate-cut sections were stained with cresyl violet (Sigma-Aldrich, St. Louis, MO. USA), luxol fast blue and sirius red stainings using standard histological protocols^29,38,39^.

### Quantification of histological stainings

#### Contusion volume

Eight coronal section from bregma + 0.6 to -4.0 mm were acquired with an Olympus BX-61 Virtual Stage microscope using a 2 × objective lens, with a pixel size of 3.49 µm. Contusion volume 1w and 5w after TBI was analysed as previously described^40^.

#### Image acquisition for histopathological analysis

Neuronal cell count, contralateral white matter quantification, collagen deposition and immunohistochemistry assays were performed 5w after TBI. Three 20 µm thick coronal sections at 0.4, 1.6, and 2.8 mm posterior to bregma were selected from each mouse brain. The entire sections were acquired with an Olympus BX-61 Virtual Stage microscope using a 20 × objective lens, with a pixel size of 0.346 µm. Acquisition was done over 10 µm thick stacks, with a step size of 2 µm. The different focal planes were merged into a single stack by mean intensity projection to ensure consistent focus throughout the sample ^33^.

#### Neuronal density

Neuronal density was performed at 1w and 5w after TBI by segmenting the cells over the entire cortex and in the corresponding contralateral hemisphere and excluding the round-shaped signal sized below the area threshold of 25 mm^2^ that is known to be associated with glial cells as reported previously^17^. Quantification was performed by Fiji software. Data were expressed as the total number of neurons quantified in the selected cortical region.

#### Contralateral white matter

White matter areas of corpus callosum (CC) and external capsula (EC) were disrupted by the focal trauma pathology in the ipsilateral hemisphere, so only the contralateral hemisphere was quantified. The whole contralateral CC was selected, and the contralateral EC was analysed up to the lower limit of the primary somatosensory cortex. The whole selected region of interest (ROI) were analysed.

#### Collagen deposition

Collagen deposition was quantified over an area included within a 300 µm radius from the contusion edge. Quantification was performed by Fiji software by segmenting the positive red signal. Data were expressed as the percentage of collagen area within the ROI.

#### GFAP, CD11b and CD68 staining

Immunohistochemistry was performed on 20 µm thick coronal sections from perfused mouse brains. The sections were incubated overnight at 4 °C with primary monoclonal antibody anti-mouse GFAP (0.5 µg/ml, 1:2000; Millipore, Billerica, MA, USA), anti-mouse CD11b (at 1w and 5w, 1.25 µg/ml, 1:800; Bio rad, Hercules, CA, USA) or anti-mouse CD68 (1.0 mg/ml, 1:200; Bio rad, Hercules, CA, USA). Biotinylated secondary antibodies (7.5 µg/ml, Vector Laboratories, Burlingame, CA, USA) were used. GFAP, CD11b and CD68 immunopositive cells were identified by reaction with 3,3 diaminobenzidine-tetrahydrochloride (DAB, Vector Laboratories, Burlingame, CA, USA) as previously described^41^. Negative control studies, without the primary antibody, were performed in parallel.

The ipsilateral cortex was analyzed over an area included within a 350 µm radius from the contusion edge. Images were analyzed using Fiji software by segmenting the positive signal. GFAP, CD11b and CD68 immunostained area were expressed as positive pixels/total assessed pixels and reported as the percentage of total stained area^18^.

### Morphological analysis

Morphological analysis was carried out on CD11b-stained Sects. 1w after TBI. Image processing was performed using Fiji software. An algorithm was created to segmentate and analyze stained cells. Briefly, images were first scaled into microns (pixel size = 0.172 × 0.172 µm). Background was subtracted and a math operation was applied so that all the gray values greater than a specified constant were replaced by the constant. The constant was defined by an operator on the basis of the best segmentation performance on pilot images and did not change throughout the experimental groups. Images were then binarized and smoothed to best fit cell shape and get rid of single positive pixels still present in the background. A further step of pixel erosion helped to achieve satisfactory cell shape fitting. To be sure to select only cells entirely present in the acquired field, cells with area > 25 µm^2^ were considered for analysis. Once segmented, the objects were measured for the following parameters: area, perimeter, Feret’s diameter (max caliper), circularity, and solidity^29,42^. Mean single cell values for each parameter were used for statistics.

### Experimental design and statistics

Mice subjected to surgery, performed by the same investigator in order to reduce variability, were randomly allocated across cages and days. Different blinded investigators evaluated mice with behavioural, histological, immunohistological and biochemical tests. Group size is of 7 defined by the formula: n = 2σ^2f.^ (α, β)/Δ^2^ (santard deviation, SD, in groups = σ, type 1 error α = 0.05, type II error β = 0.2, percentage difference between groups Δ = 30). Standard deviation to be used in the formula was calculated based on a pilot experiment to assess PTX3 staining in cortex 5w after TBI, resulting in σ = 21, thus yielding n = 7.742. Groups were compared by analysis of variance and post hoc testing as indicated in each figure legend. A parametric or nonparametric test was selected after the Kolmogorov–Smirnov test for normality to assess whether the data for the groups were normally distributed. The constancy of the variances was checked by the Bartlett test and, if not satisfied, a Welch correction applied to the test. Statistical analysis was performed with the standard software package GraphPad Prism (GraphPad Software Inc., San Diego, CA, USA, version 7.0); p values lower than 0.05 were considered significant.

### Ethics approval

Procedures involving animals and their care were conducted in conformity with institutional guidelines in compliance with national and international laws and policies. The experimental protocols were approved by Ethical Committee at the Istituto di Ricerche Farmacologiche Mario Negri IRCCS and by Italian Ministry of Health (prot. 9F5F5.81, authorization nº 753/2017-PR). The protocols and details of this report are in accordance with ARRIVE guidelines (http://www.nc3rs.org.uk/page.asp?id=1357) and the check list is provided as Supplementary file.

## Results

**Figure 1:**
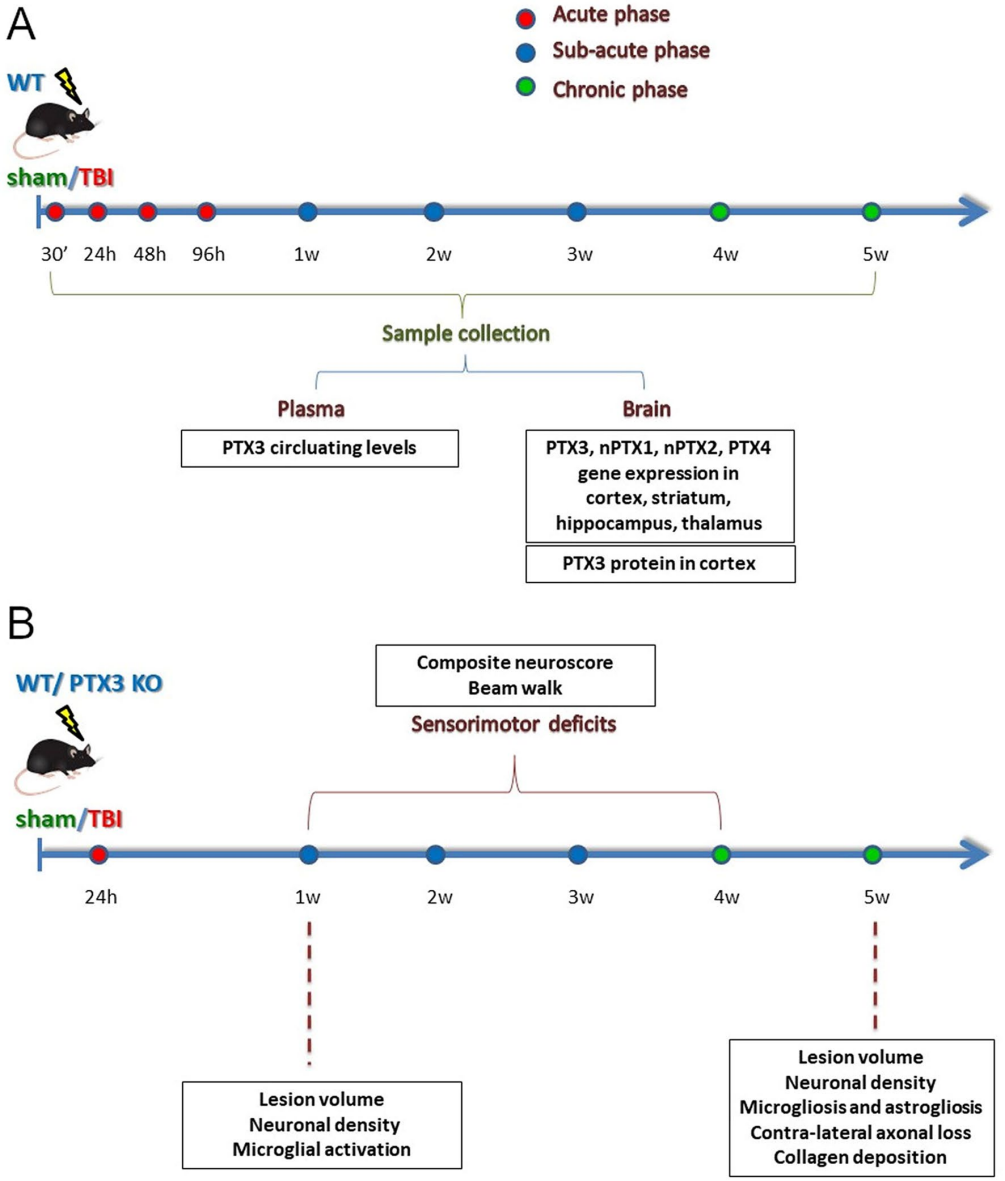
Experimental Plan. (**A**) WT mice underwent TBI or sham operation. TBI mice were sacrificed at different time points after surgery and plasma, whole brain or brain areas including cortex, striatum, thalamus and hippocampus were collected. PTX3 plasmatic levels were measured by ELISAs (naive; sham: 24 h, 5w; TBI: 30′, 24 h, 48 h, 96 h, 1w, 2w, 3w, 5w). PTX3 presence (sham: 5w; TBI: 30′, 24 h, 48 h, 96 h, 1w, 2w, 3w, 4w, 5w) and co-localization with neutrophils (Elastase; 48 h), neurons (NeuN; 1w), astrocytes (GFAP; 1w), microglia (CD11b; 1w), endothelial cells (CD31; 1w) and fibrin(ogen) (1w,2w) was studied by immunofluorescence assay on perfused brains. nPTX1, nPTX2, PTX3, PTX4 gene expression analysis was done on snap frozen brain areas by RT-qPCR (sham: 24 h, 96 h, 1w, 2w, 5w; TBI: 24 h, 96 h, 1w, 2w, 5w). (**B**) WT or PTX3 KO underwent CCI or sham operation. Sensorimotor deficits were assessed by composite neuroscore and beam walk tests on a weekly basis for four weeks after TBI. Brains from both strains were harvested for histopathological analysis: lesion volume and neuronal density with cresyl violet staining (1w; 5w); collagen presence with sirius red staining (5w); contra-lateral white matter loss with luxol fast blue staining (5w); astrogliosis (GFAP; 5w), microgliosis (CD11b/CD68; 5w) and shape descriptors microglia (CD11b; 1w) with immunohistochemistry.

This study was carried out according to the plans in Fig. 1. First, PTX3 was analyzed at different time points after TBI or sham surgery for its circulating levels or brain gene expression and protein presence (Fig. 1A). Next, in order to define PTX3 role in TBI pathophysiology we investigated behavioral deficits and brain damage progression over 5 weeks after TBI comparing WT to PTX3 KO mice (Fig. 1B).

### Pentraxin family gene expression in brain areas over 5 weeks after TBI

**Figure 2:**
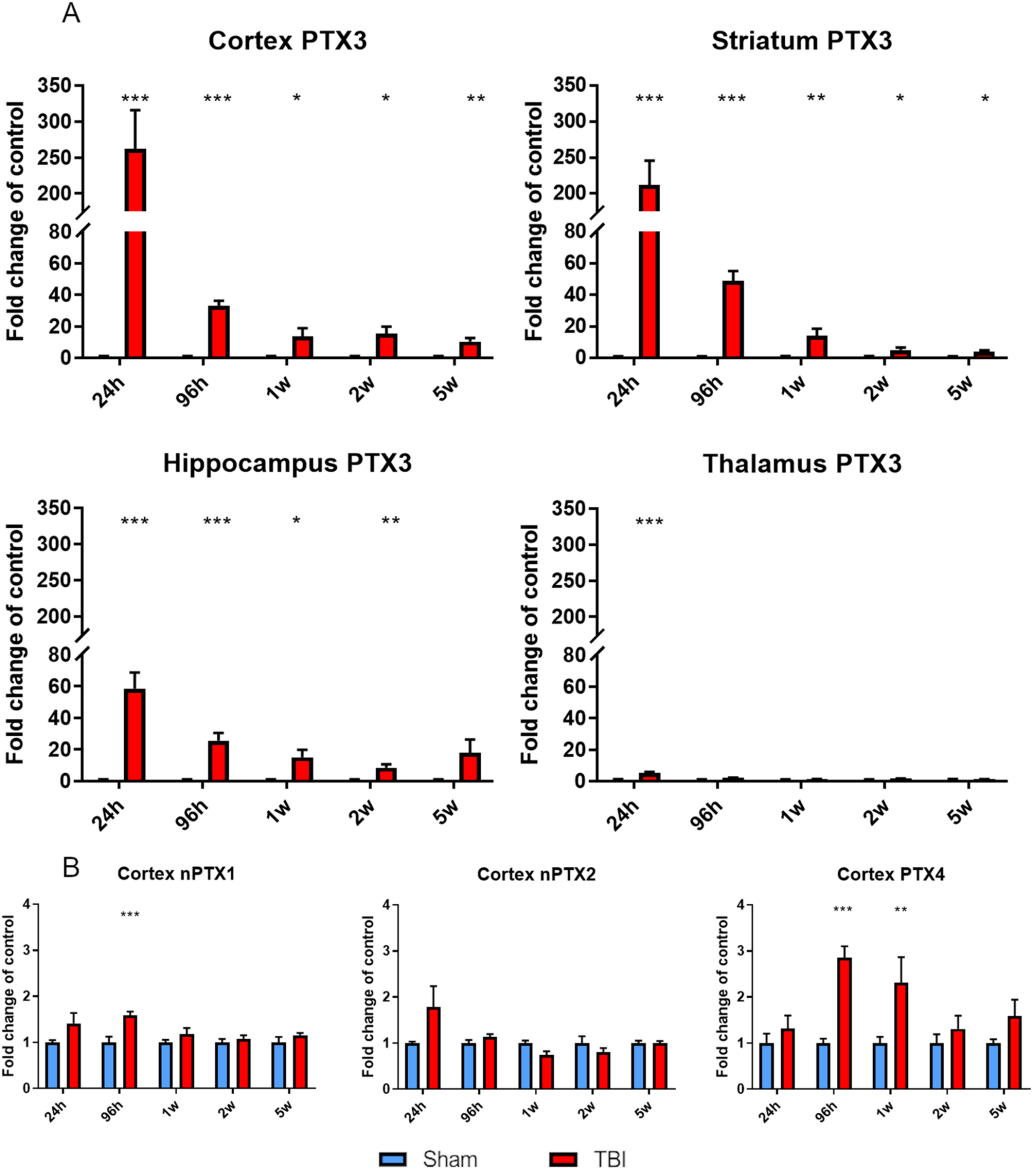
PTX3, nPTX1, nPTX2 and PTX4 mRNA expression in lesioned brain areas. (**A**) PTX3 was upregulated in the ipsilateral cortex, striatum, hippocampus and thalamus early (24 h) and up to 2w (hippocampus) or 5w (cortex and striatum) after TBI compared to sham mice. (**B**) nPTX1 and PTX4 cortical expression were significantly increased 96 h after TBI and up to 1w (PTX4) compared to sham, while nPTX2 expression was uneffected. Data is presented as mean ± SEM, n = 6–8. For PTX3 in thalamus and nPTX1 and PTX4 in cortex computations assume that all rows are sampled from populations with the same scatter SD. Multiple t-test followed by Holm-Sidak post hoc test, *adjusted *p* < 0.05; **p < 0.01; ***p < 0.001 vs sham.

mRNA expression of *PTX3* and that of other members of the long pentraxin family that are expressed in the brain^43–45^ were measured in cortex, striatum, hippocampus and thalamus obtained from TBI and sham mice by RT-qPCR. *PTX3* was upregulated in the ipsilateral cortex, striatum, hippocampus and thalamus early (24 h; 261.42 ± 54.44, 211.62 ± 33.81, 58.52 ± 10.25 and 5.13 ± 0.78 fold-change than sham ± standard error mean, SEM, respectively) and up to 2w (hippocampus; 17.82 ± 8.55) or 5w (cortex and striatum; 10.35 ± 2.21 and 3.85 ± 0.93 respectively) after TBI compared to sham mice (Fig. 2A). *nPTX1* and *PTX4* cortical expression were significantly increased 96 h after TBI (1.59 ± 0.08 and 2.31 ± 0,56, respectively) and up to 1w (*PTX4*; 2.86 ± 0.25) compared to sham, while *nPTX2* expression was unaffected (Fig. 2B). Finally, *nPTX1*, *nPTX2* and *PTX4* gene expression in striatum, thalamus and hippocampus did not change at any time points after TBI compared to sham mice (Supplementary Fig. 1). *CRP* and *SAP* were not expressed in either control or TBI group (Supplementary Table 1).

### PTX3 plasma levels and brain presence over 5 weeks after TBI

**Figure 3:**
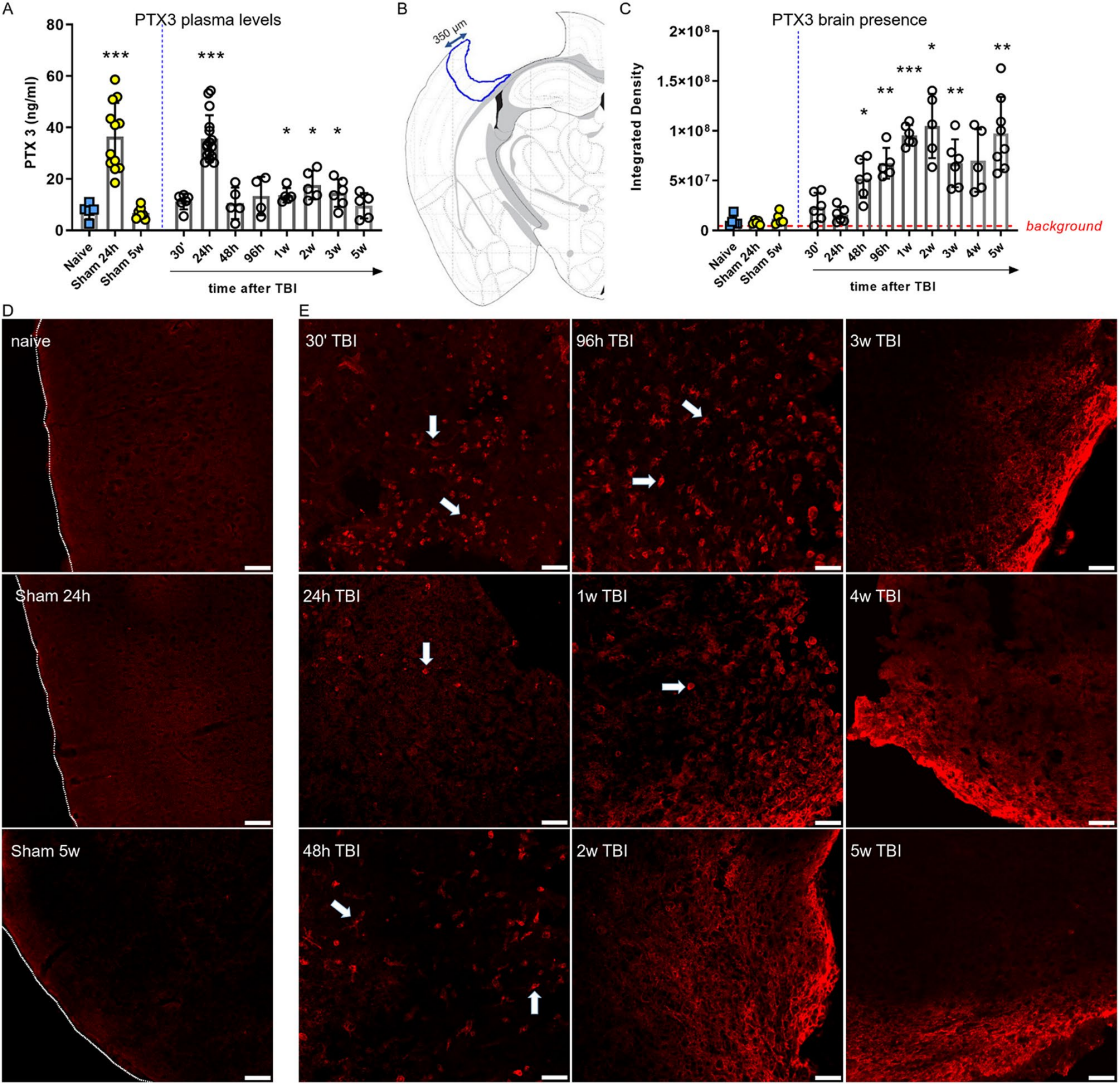
PTX3 protein presence in plasma and in cortex up to 5 weeks after TBI. (**A**) At 24 h plasmatic PTX3 levels increased in both sham and TBI mice. PTX3 levels were significantly higher than naive from week 1 to week 3. Data is presented as mean ± SEM, n = 4 (TBI 96 h), n = 5 (naive, TBI: 48 h, 1w, 2w, 5w), n = 6 (TBI 30′, 3w), n = 16 (sham 24 h), n = 7 (sham 5w), n = 16 (TBI 24 h). Unequal variances per Bartlett’s test, t-test with Welch correction, *p < 0.05, ***p < 0.001, vs naive. (**B**) PTX3 presence in cortex was evaluated by immunofluorescence and quantified in a ROI placed in the il-cortex within the first 350 μm from the edge of the contusion (**B**). (**C**) PTX3 increased starting from 48 h and reached its maximum at 2 weeks after TBI. Data is presented as mean ± SEM, n = 5–6. Unequal variances per Bartlett’s test, one-way ANOVA with Welch correction, *p < 0.05, **p < 0.01, ***p < 0.001, vs naive. (**D**) Representative confocal microscopy images showing that PTX3 was not present in naïve or sham mice (sacrificed at 24 h and 5w). Tracings indicate the cortex edge, scale bars = 50 µm. (**E**) Representative confocal microscopy images showing PTX3 (red) presence next to cell-like shape (arrows) up to 1 week after injury, while it was located mainly extracellularly at longer time points (from 2 to 5w after TBI). Scale bars = 50 µm.

At 24 h after TBI, plasmatic PTX3 levels sharply increased in both TBI and sham-operated mice (35.69 ± 2.27 vs 36.41 ± 3.80, ng/mL ± SEM, Fig. 3A), implying an effect of the surgical procedure—not selectively associated with TBI induction. Analyzing further time points from surgery, PTX3 levels were significantly higher than naïve (7.51 ± 1.37) from week 1 to week 3 (13.63 ± 1.25, 17.63 ± 2.51 and 14.5 ± 2.18, respectively). Brain PTX3 levels were investigated in cortex after TBI by immunofluorescence and quantified under a ROI placed in the ipsilateral cortex within the first 350 μm from the edge of the contusion (left panel, Fig. 3B). PTX3 increased starting from 48 h (52.02 × 106 ± 79.12 × 105, ID ± SEM), reached its maximum at 2w (10.49 × 107 ± 14.41 × 106) and up to 5w after TBI (97.63 × 106 ± 12.89 × 106) compared to naive mice (9.05 × 106 ± 24.87 × 105, Fig. 3C). In contrast with the circulating levels, brain PTX3 was not affected by the surgical procedure, i.e. at 24 h after the sham procedure PTX3 was not seen in the brain (Fig. 3D). Thus PTX3 presence in the brain, observed starting from 48 h, was specifically caused by TBI. It appeared in cellular structures (arrows in Fig. 3E) up to 1 week after TBI, while it was located mainly extracellularly at longer time points (from 2 to 5w after TBI, Fig. 3E).

### Confocal analysis of PTX3 presence in the contused cortex at different time points after TBI

**Figure 4:**
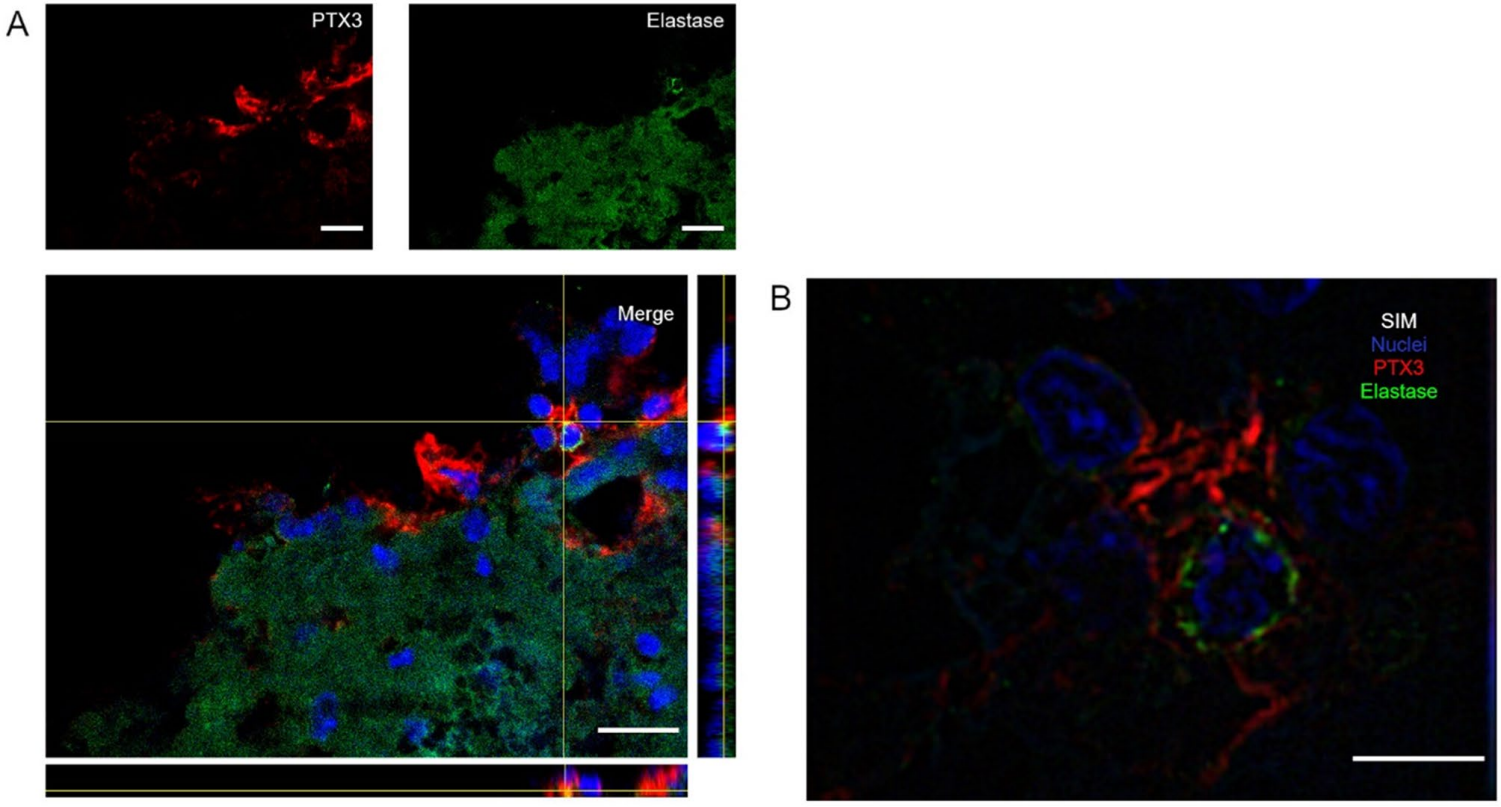
Confocal analysis of PTX3 localization with neutrophils in the contused cortex 48 h after TBI. (**A**) PTX3 (red) was localized in proximity of neutrophils (Elastase, green). Scale bar = 20 µm. (**B**) High magnification obtained using SIM showing PTX3 with branches surrounding a neutrophil, likely indicating its presence on NETs. Scale bar = 5 µm.

Starting from the notion that PTX3 has been reported in neutrophil granules^46^, we assessed the possible co-localization between neutrophil marker elastase and PTX3 by immunofluorescence. PTX3 was localized in proximity of neutrophils 48 h after TBI (Fig. 4A). By SIM, we observed PTX3 with branches surrounding a neutrophil, likely indicating its presence on extracellular NETs (Fig. 4B). Immunofluorescent controls showed no signal in PTX3 KO mice and in WT stained without the primary antibody, confirming the staining specificity (Supplementary Fig. 2).

**Figure 5:**
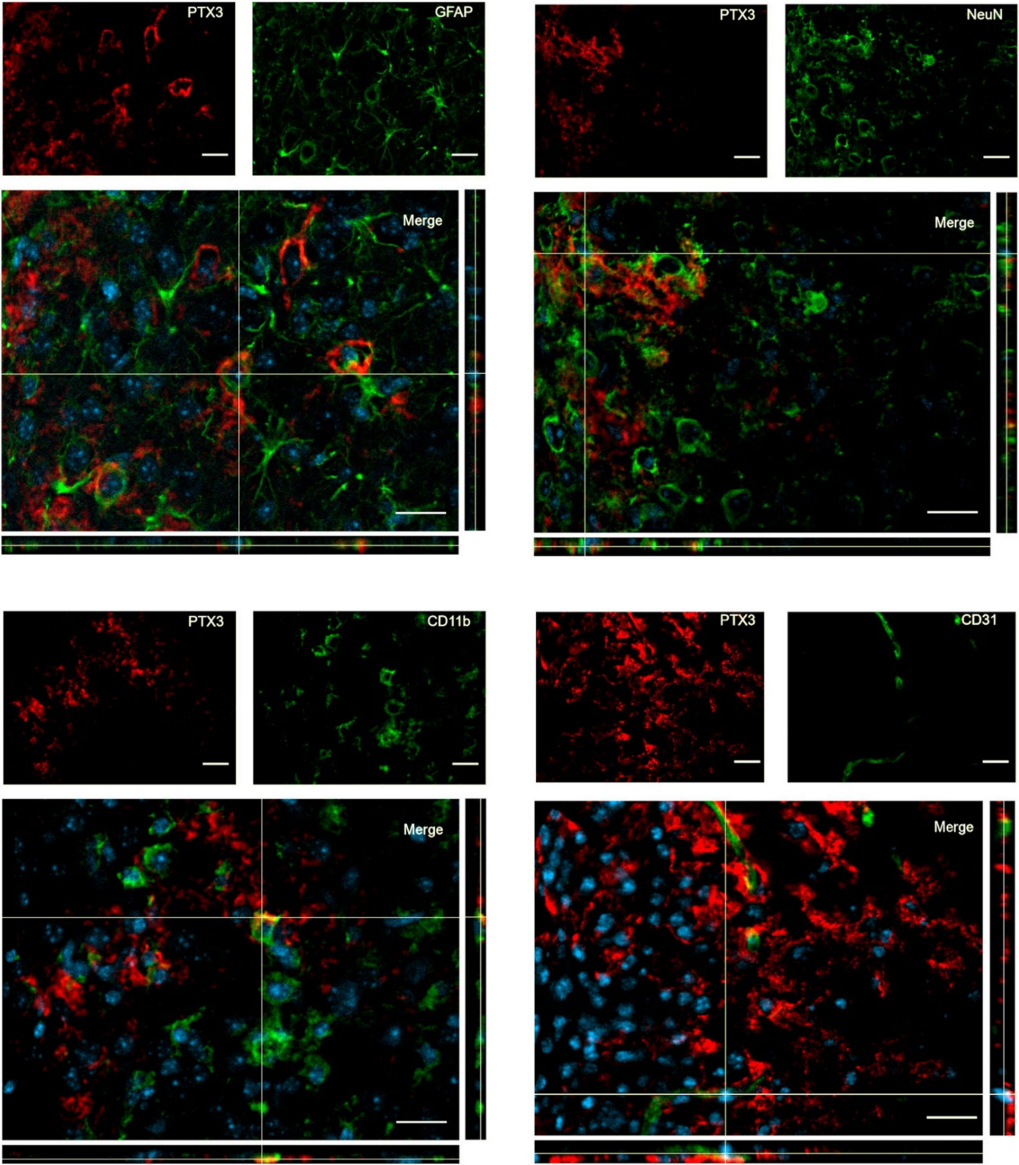
Confocal analysis of PTX3 presence in the contused cortex 1 week after TBI. PTX3 (red) was present next to but did not co-localize with astrocytes (GFAP, green), neurons (NeuN, green), microglia (CD11b, green) and endothelial cells (CD31, green). Nuclei are in blue. Images are representative of at least two independent experiments. Scale bar = 20 µm.

We next evaluated PTX3 localization in relation to different cell populations present in the injured tissue including astrocytes, neurons, microglial and endothelial cells, 1 week after TBI, a time point at which PTX3 signal appears next to cell-like structures (Fig. 3C). PTX3 signal was present next to but did not co-localize with any of these cell populations in the contused cortex (Fig. 5).

**Figure 6:**
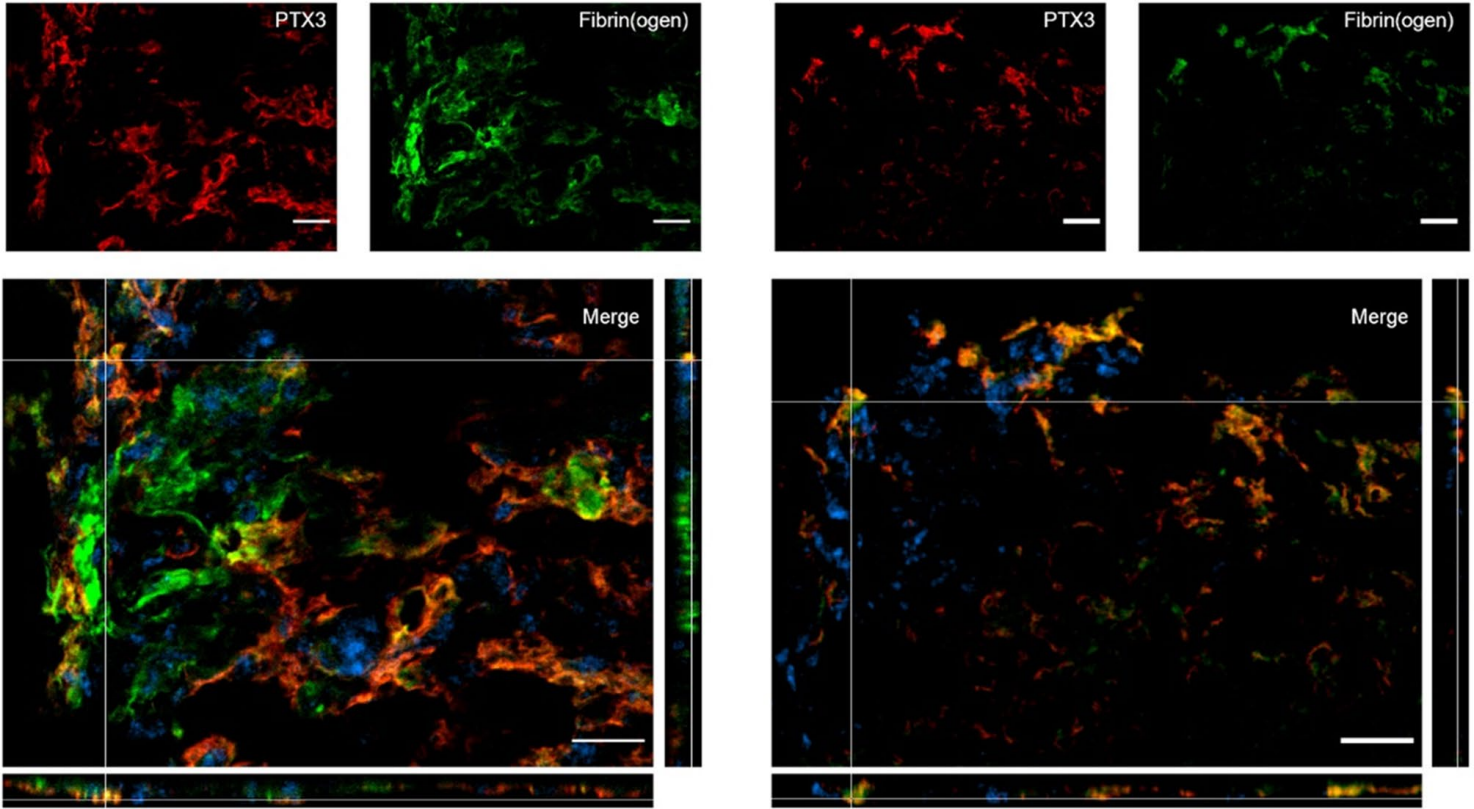
PTX3 co-localization with fibrin(ogen) in the contused cortex 1 and 2 weeks after TBI. PTX3 (red) co-localized with fibrin(ogen) (green) at 1w (left) and 2w (right) after TBI. Nuclei are in blue. Images are representative of at least two independent experiments. Scale bar = 20 µm.

We then addressed extracellular presence of PTX3 at 1w and 2w after TBI and investigated possible connections with fibrin deposits. At both time points we found a strong co-localization between PTX3 and fibrin(ogen) ( Fig. 6).

A summary of the results of PTX3 expression and presence after brain injury is reported in Table 1.

**Table 1:** Summary table of brain PTX3 mRNA expression, protein presence and localization over time after TBI.

PTX3	Naive	Sham	TBI 30’	TBI 24 h	TBI 48 h	TBI 96 h	TBI 1w	TBI 2w	TBI 3w	TBI 4w	TBI 5w
**Gene expression**											
mRNA: cortex	–	Control group	–	+ + +	–	+ + +	+	+ +	–	–	+
mRNA: striatum	–	Control group	–	+ + +	–	+ + +	+ +	+	–	–	=
mRNA: hippocampus	–	Control group	–	+ + +	–	+ +	+ +	+	–	–	+ +
mRNA: thalamus	–	Control group	**–**	+	–	=	=	=	–	–	=
**Protein presence**											
Plasma	Control group	+ + + (24 h) = (5w)	+	+ + +	+	+	+ +	+ + +	+ +	–	=
Brain cortex	Control group	= (24 h) = (5w)	=	=	+	+ +	+ + +	+ + +	+ + +	+	+ +
**Protein localization**											
Neutrophils	–	–	–	–	Next to	–	–	–	–	–	–
Astrocytes	–	–	–	–	–	–	Next to	–	–	–	–
Microglia	–	–	–	–	–	–	Next to	–	–	–	–
Neurons	–	–	–	–	–	–	Next to	–	–	–	–
Endothelial cells	–	–	–	–	–	–	Next to	–	–	–	–
Fibrin(ogen)	–	–	–	–	–	–	Co-localization	Co-localization	–	–	–

The long-lasting pattern of activation of PTX3 in brain and blood following TBI supports the hypothesis that PTX3 contributes to the progression of the lesion with effects varying over time. Increases versus respective controls are calculated according to quartiles and correspond to: = control group; + low increase; +  + intermediate increase; +  +  + high increase.

### Effects of PTX3 genetic depletion on TBI long-term outcomes

**Figure 7:**
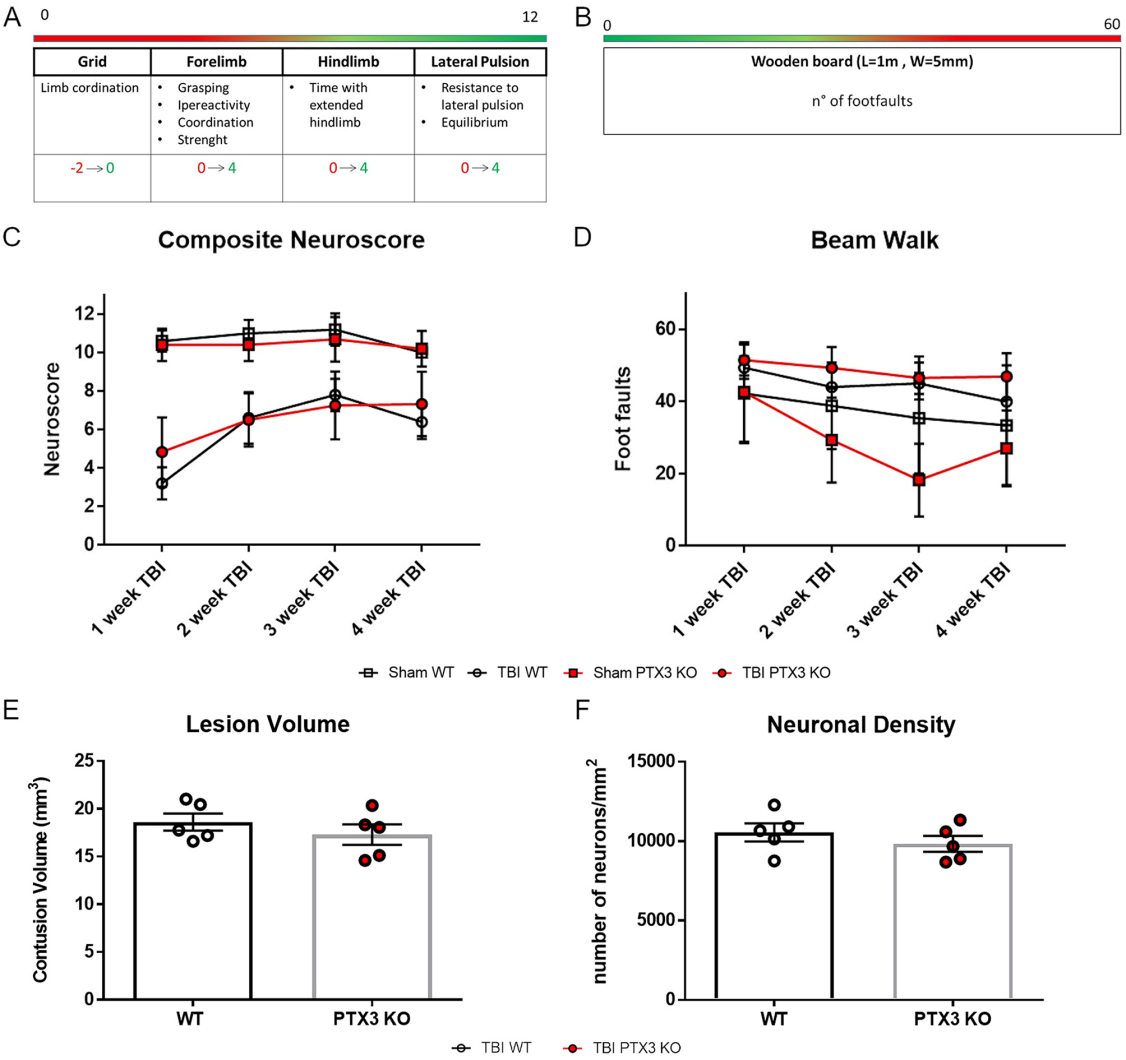
PTX3 depletion did not affect TBI outcome. (**A**, **B**) Sensorimotor function was assessed over 4 weeks after sham injury or TBI using Composite neuroscore (**A**, worst score = 0) and Beam walk (**B**, worst score = 60) tests in WT and PTX3 KO mice. (**C**, **D**) No difference was observed in post-traumatic sensorimotor deficits between the two strains. Data is presented as mean ± SEM, n = 5 (Sham/TBI WT), n = 10 (Sham PTX3 KO), n = 12 (TBI PTX3 KO). Two-way ANOVA for repeated measures followed by Bonferroni post hoc test = ns. Five weeks after TBI, neither the lesion volume (**E**) nor the neuronal density (**F**, neurons per mm^2^) in the lesioned cortex varied between WT and PTX3 KO mice. Data is presented as mean ± SEM, n = 5 (TBI WT/PTX3 KO). Unpaired t-test = ns (**E**).

Sensorimotor function was assessed over 4 weeks after sham injury or TBI using Neuroscore (Fig. 7A) and Beam Walk (Fig. 7B) tests in WT and PTX3 KO mice. No difference was observed in post-traumatic sensorimotor deficits between the two strains (Fig. 7C-D). We then assessed lesion volume and neuronal density at 5 weeks after TBI by cresyl violet staining. We observed an extensive macroscopic area of cortical tissue loss, extending rostrocaudally from bregma + 0.4 to -3.6 mm, both in WT and PTX3 KO animals, without differences in the lesion volume between the two genotypes (Fig. 7E). Also, the neuronal density in a cortical region, traced at a distance of 350 μm from the contusion edge, did not differ between WT and PTX3 KO mice (Fig. 7F).

### Effects of PTX3 depletion on short and long-term brain inflammatory processes

**Figure 8:**
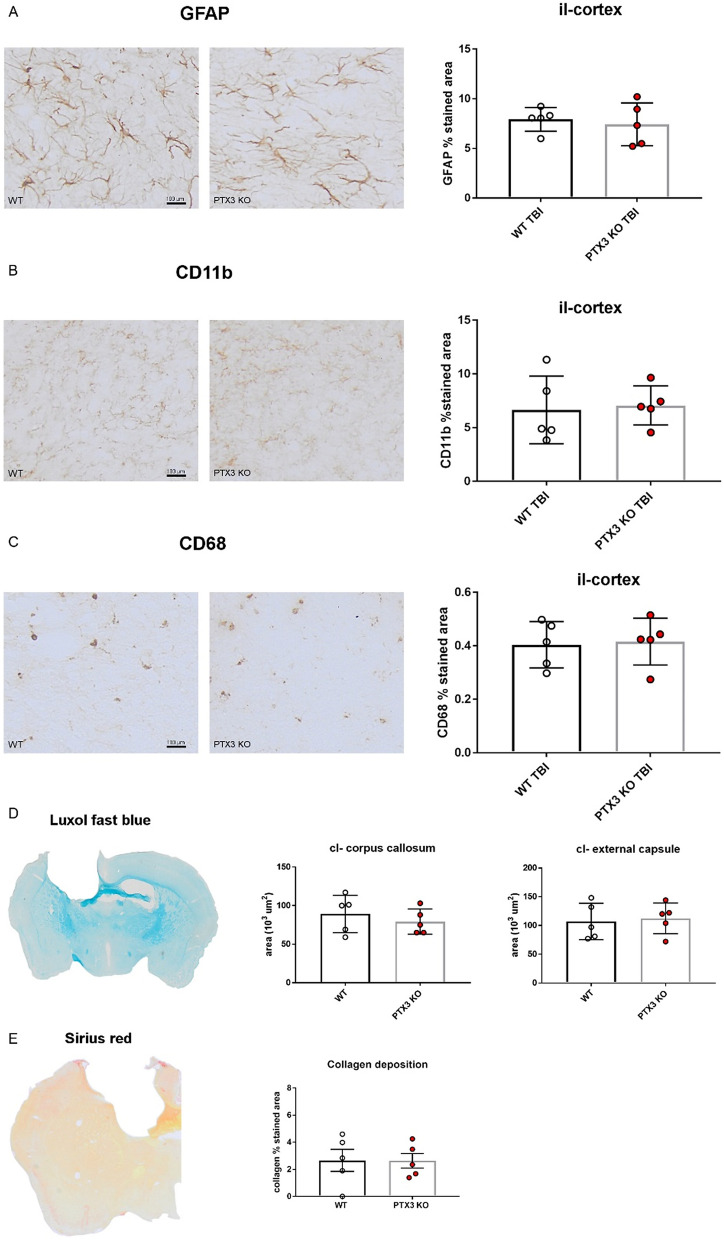
PTX3 depletion did not modify brain inflammatory response at 5 weeks after TBI. Astrocytes (**A**, GFAP), brain myeloid cells (**B**, CD11b; **C**, CD68) and collagen deposition (**E**, by sirius red) were quantified in the quantified in the ipsilateral (il)-cortex within the first 350 μm from the edge of the contusion showing no difference between WT and PTX3 KO. Axonal loss in contralateral (cl)-corpus callosum and external capsule (**D**, by luxol fast blue) did not differ between the two strains. Data is presented as mean ± SEM, n = 5 (TBI WT/PTX3 KO). Unpaired t-test = ns.

We also analyzed brains obtained from WT and PTX3 KO TBI mice sacrificed at 1 week after TBI, in order to evaluate acute effects of PTX3 depletion. Neither the lesion volume (12.82 ± 1.055 vs 14.44 ± 1.164, mm^3^ ± SEM) nor the neuronal density (3118 ± 117.9 vs 3163 ± 247.4, number of neurons/mm^2^ ± SEM) showed differences between the two strains (Supplementary Fig. 3A,B). Since microglial morphology may be associated with their function, we next analyzed few shape descriptors^42^, observing no differences between WT and PTX3 KO mice (Supplementary Fig. 3C). In the chronic phase (5 weeks after TBI) we measured astrogliosis and microglial activation, quantifying GFAP, CD11b and CD68 immunopositive area at the edge of the contusion area. No differences were present between WT and PTX3 KO mice for GFAP (Fig. 8A), CD11b (Fig. 8B) and CD68 (Fig. 8C) stainings. We also assessed the axonal loss by luxol fast blue staining. The stained hemisphere did not show any difference between WT and PTX3 KO mice in either the corpus callosum or the external capsule (Fig. 8D). In different models of tissue damage, PTX3 deficiency has been associated with enhanced collagen deposition^47^. Therefore, we measured collagen deposition within the first 350 μm from the edge of the contusion by sirius red staining. No difference was present between WT and PTX3 KO mice (Fig. 8E).

## Discussion

The present study originally shows that, following TBI: (1) PTX3 circulating levels are increased in the sub-acute phase, starting 1 week and up to 3 weeks post-injury; (2) in the brain, PTX3 gene expression is increased in response to TBI, as early as 24 h and up to 5 weeks after the primary insult; (3) PTX3 protein is present in the injured cortex, specifically in association with NETs in the acute phase, and bound to fibrin(ogen) deposits in the sub-acute phase; (4) the brain gene expression of long pentraxin nPTX1 and PTX4 is also increased after TBI. Despite the consistent presence of PTX3 at different time points after TBI, its genetic depletion did not affect TBI outcome, as assessed up to 5 weeks post-injury, in terms of sensorimotor deficits, lesion size or inflammatory markers.

Although weakly expressed in basal conditions in the central nervous system (CNS), *ptx3* gene transcription may be induced in different brain cells—among which glial cells, mononuclear phagocytes and endothelial cells—in response to a wide range of pro-inflammatory stimuli, including IL-1β, TNF-α, toll-like receptor (TLR) ligands in addition to microbial components^43,48^. We actually found a strong activation of *PTX3* mRNA expression compared to control mice in brain areas adjacent to TBI lesion core, namely cortex and striatum, occurring at early time points (24 h) and lasting up to 5w after insult. Additional brain areas next to the lesion core, such as hippocampus and thalamus, also showed *PTX3* overexpression after TBI, although to a lower extent. These observations indicate that PTX3 participates in brain acute phase responses elicited by TBI in both lesion core and surrounding areas. *PTX3* mRNA is still present in sub-acute and chronic responses, but exclusively in areas adjacent to the lesion core, supporting the hypothesis that, at this stage, PTX3 may have a role in the formation of the gliotic scar and/or in healing processes, as previously shown in other models or tissues^7,12^.

Other members of the pentraxin family, namely the long pentraxins *nPTX1* and *PTX4*, but not *nPTX2* (nor the short pentraxins *CRP* and *SAP*), were also upregulated, although to a lower extent compared to *PTX3*, in the cortex at the early phases after TBI, suggesting the involvement of nPTX1 and PTX4 in the acute brain responses to TBI. nPTX1 was reported to have a role in neuronal death after ischemia in vitro^49^ and in vivo^50^ suggesting its involvement in the traumatic pericore tissue, an area subjected to post-injury hypoxia^22^. At variance, scanty information is available on PTX4^45^ which however shows a clearcut upregulation after TBI.

Available data show that circulating PTX3 levels increase rapidly in response to infections and cardiovascular diseases^3,6^. Elevated PTX3 plasma levels are recognized as independent predictors of mortality at three months after acute myocardial infarction^51,52^. Circulating PTX3 is also associated with plaque vulnerability/rupture^53–55^, incidence of heart failure, cardiac arrest^6,56–58^ and hypoxic respiratory failure^59^, suggesting a role as biomarker of cardiovascular risk^48^. In the CNS, PTX3 role as biomarker was proposed also in acute brain injury. Specifically, PTX3 was been identified as a novel and independent prognostic marker in ischemic stroke in both human^60^ and mice^12^. Finally, PTX3 protein levels increased in human cerebrospinal fluid (CSF) early (48 h) after subarachnoid hemorrhage (SAH), with a second peak of expression in the following 48–96 h associated with increased occurrence of vasospasm^61^. In TBI patients, serum PTX3 levels, measured within 72 h of hospital admission, were reported to increase significantly after severe injury and to be independently associated with hospital mortality^23^. In our TBI model we show a persistent increase in PTX3 circulating levels. We observed a sharp increase in plasma PTX3 at 24 h post-injury which however appeared to be non-specific and possibly due to PTX3 release following skin injury, as previously demonstrated at the same time-point by Doni et al.^7^. At later time points we observed a clear-cut and long-lasting increase in plasma PTX3 compared to non-TBI controls, starting from 1w after TBI. At least part of PTX3 systemic increase can be due to its release by brain cells into cerebral blood flow through the injured blood brain barrier (BBB). Leukocytes may be an additional source of PTX3, thus potentially contributing to PTX3 circulating levels by protein release in blood^62^.

TBI induces neutrophil recruitment to the site of damage, making them an important source of PTX3 in the traumatic brain^63^. In particular, neutrophils act as a reservoir of ready-made PTX3, stored as glycosylated form in specific granules^48^, and rapidly released to sites where tissue damage (or microbial stimulation) occurs^64^. PTX3 is complexed with components of NETs, a structure involved in inflammation, phagocytosis and coagulation^46,48,65,66^. PTX3 function in NETs is known in non-sterile inflammation, where it brings the NET component proteins into close proximity with the pathogens to enhance pathogen clearance^65^. We report for the first time in TBI that PTX3 is present in the mouse brain parenchyma in acute phases after injury and is associated with NETs. The exact functions associated with NET-bound PTX3 in sterile inflammation, like that associated with TBI, still need clarification. PTX3 was reported to reduce neuroinflammation at early time points after intrastriatal lipopolysaccharide (LPS) administration or after stroke by dampening neutrophil recruitment into the brain^14^. Other potential roles of PTX3 in the context of acute brain injury comprise NET-mediated thrombosis^67–69^. We show here that PTX3 was present in cortex next to astrocytes, neurons, microglia and endothelial cells in the sub-acute phase, suggesting a possible release from these cells with a paracrine function. The expression of PTX3 by each of these brain cell populations has been associated with a specific function in response to brain damage. Namely, PTX3 in astrocytes has been related to BBB integrity in the acute phase of stroke^13,43^; in neurons it conferred resistance to neuronal damage at sub-acute phases after seizures^10^ and mediated neurogenesis and angiogenesis after stroke^11,70^. At variance with the protective functions described above, in cultured endothelial cells PTX3 was released in response to inflammatory stimuli^71–74^ acting as critical determinant of the endothelial dysfunction^48^. In addition, it promoted glial proliferation and glial scar formation after stroke, thus possibly limiting the recovery of synaptic plasticity after acute brain injury^12^.

We found that PTX3 co-localized with fibrin deposits starting from the sub-acute phase up to the chronic phase after TBI. In fact, PTX3 may bind fibrinogen and/or fibrin and plasminogen and increase plasmin-mediated pericellular fibrinolysis^7^, an action related to its role in extracellular matrix remodeling. In fact PTX3 can target the extracellular matrix component fibrinogen, via its N-terminal domain ^75^, and promote wound healing by favoring fibrinolysis as shown in different models of tissue damage^7,47,76,77^, a function dependent on the acidic microenvironment generated by tissue injury^7^. Thus, PTX3 may lead to fibrinolysis and gliotic scar resolution with subsequent improvement in the injury outcome. Fibrinogen extravasation has been observed in post-mortem human brain months and even years after a single moderate-severe TBI^78^. Parenchymal deposition of fibrinogen is significantly increased following acute TBI and associated with a proinflammatory, pro-phagocytic microglial/macrophage phenotype, suggesting a role in augmenting and sustaining an inflammatory state in human TBI that impacts negatively on neuronal density and, potentially, axonal survival^79^.

The analysis of PTX3 mRNA and protein expression reveals that mRNA increases 24 h after TBI likely indicating protein production later on (48 and 96 h). The subsequent gene expression increase (96 h) is likely to result in enhanced protein production at sub-acute times (1w and 2w), when it reaches its maximum. At these time points PTX3 appears next to brain cells and also in relation to extracellular matrix fibrin(ogen). The protein parenchymal localization in the extracellular matrix follows the lower but still present PTX3 gene expression up to chronic phases. The acid environment present in the lesioned brain parenchyma may support the persistent presence of PTX3, and its involvement in tissue remodelling during sub-acute and chronic phases^7^.

An obvious approach was to assess TBI outcome in PTX3 depleted mice, according to the protocol for evaluating the injury evolution in our experimental model^18,33,40^. PTX3 depleted mice lacked a clear-cut phenotype compared to WT up to 5w after TBI. We can envisage a few possible scenarios to account for this lack of phenotype. First, PTX3 may subserve multiple functions throughout different phases of injury evolution, i.e. acute inflammatory functions vs. chronic reparative actions, finally resulting in a balanced phenotype. To explore possible time-dependent roles of PTX3 in TBI physiopathology, we assessed lesion size and neuronal viability at sub-acute phase (1w) after TBI in WT and PTX3 KO, finding no differences. This result does not directly support a prevalent inflammatory role of PTX3 in the early stages post-injury, however the multiple functions of PTX3 and its different interactions with injury-related molecules in TBI brains cannot exclude that the lack of phenotype of PTX3 KO mice may be due to a balance between damaging and protective effects. As an alternative hypothesis, PTX3 may have a bystander role in TBI, thus not actively participating to injury progression, in line with what hypothesized for a chronic pulmonary pathology^80,81^. However, since TBI elicits acute inflammation, the bystander role seems less likely compared to other hypothesis.

For instance, a possible scenario would be that brain long pentraxins could compensate for the absence of PTX3 in KO mice. Actually they share a degree of identity with PTX3, namely 21.2% with nPTX1 and 19.7% with PTX4 (sequence alignment done with Uniprot, www.uniprot.org). It may also be possible that these long pentraxins counteract PTX3 action in TBI progression, especially interfering with PTX3-associated synaptogenesis, a process that involves the remodeling of the extracellular matrix by several mediators, i.e. tumor necrosis factor-induced protein-6 (TSG6), astrocytes-secreted thrombospondins (TSPs) and β1 integrin^82,83^.Unfortunately, the lack of information available on the function of these pentraxins in the brain does not presently allow to substantiate any of these hypothesis.

## Conclusion

The long-lasting pattern of activation of PTX3 in brain and blood supports a specific involvement in TBI. The lack of a clear-cut phenotype in PTX3 KO mice may depend on the different roles of this protein, possibly involved in inflammation early after injury and in repair processes later on, and/or on the presence of brain pentraxins compensating for its absence. Available data obtained in different models of injury and inflammation, show that recombinant PTX3 administered in sub-acute phases reduces inflammation and boosts tissue reparative processes^7^. As such, our work may offer reasons to administer PTX3 to alleviate sub-acute pathological *sequelae* after TBI.

## Supplementary Information

Supplementary Information.

## Data Availability

The data sets generated and/or analysed during the current study are available in the Figshare repository, 10.6084/m9.figshare.13019081.

## Abbreviations

TBI: Traumatic brain injury
NETs: Neutrophils extracellular traps
nPTX1: Neuronal pentraxin 1
nPTX2: Neuronal pentraxin 2
PTX3: Pentraxin 3
PTX4: Pentraxin 4
CRP: C-reactive protein
SAP: Serum amyloid P-component
CCI: Controlled cortical impact
WT: Wild-type
PTX3 KO: PTX3 depleted
EDTA: Ethylenediaminetetraacetic acid
PBS: Phosphate buffer saline
ELISA: Enzyme-linked immunosorbent assay
NA: Numerical aperture
FHS: Fetal horse serum
GFAP: Glial fibrillary acid protein
GIMP: GNU Image Manipulation Program
CC: Corpus callosum
EC: External capsula
ROI: Region of interest
DAB: Diaminobenzidine-tetrahydrochloride
SD: Standard deviation
SEM: Standard error mean
ID: Integrated density
SIM: Structured illumination microscopy
TLR: Toll-like receptor
CSF: Cerebrospinal fluid
SAH: Subarachnoid hemorrhage
BBB: Blood brain barrier
LPS: Intrastriatal lipopolysaccharide
