# Supplementary material for: Long pentraxin PTX3 is upregulated systemically and centrally after experimental neurotrauma, but its depletion leaves unaltered sensorimotor deficits or histopathology
Source: Sci Rep. 2021 May 5;11:9616. doi: 10.1038/s41598-021-89032-7 (PMC8100171; doi:10.1038/s41598-021-89032-7)
Supplement: Supplementary file 1 — Supplementary Information. [file 41598_2021_89032_MOESM1_ESM.docx]

**Long pentraxin PTX3 is upregulated systemically and centrally after experimental neurotrauma, but its depletion leaves unaltered sensorimotor deficits or histopathology**

Marco Oggioni^1^, Domenico Mercurio^1^, Denise Minuta^1#^, Stefano Fumagalli^1^, Katarzyna Popiolek-Barczyk^3^, Marina Sironi^2^, Agata Ciechanowska^3^, Stefania Ippati^1##^, Daiana De Blasio^1^, Carlo Perego^1^, Joanna Mika^3^, Cecilia Garlanda^2^ and Maria-Grazia De Simoni^1*^

*^1^ Istituto di Ricerche Farmacologiche Mario Negri IRCCS, Milan, Italy;*

*^2^ IRCCS- Humanitas Research Hospital IT;*

*^3^ Department of Pain Pharmacology, Maj Institute of Pharmacology Polish Academy of Sciences, Krakow, Poland*

^#^ Present address: San Raffaele Telethon Institute for Gene Therapy (SR-Tiget), San Raffaele Hospital, Milan 20132, Italy.

^##^ Present address: San Raffaele Scientific Institute, San Raffaele Hospital, Milan 20132, Italy.

***Corresponding author**:

Maria-Grazia De Simoni,

Istituto di Ricerche Farmacologiche Mario Negri IRCCS

via Mario Negri 2, Milan 20156, Italy.

**Email**: desimoni@marionegri.it


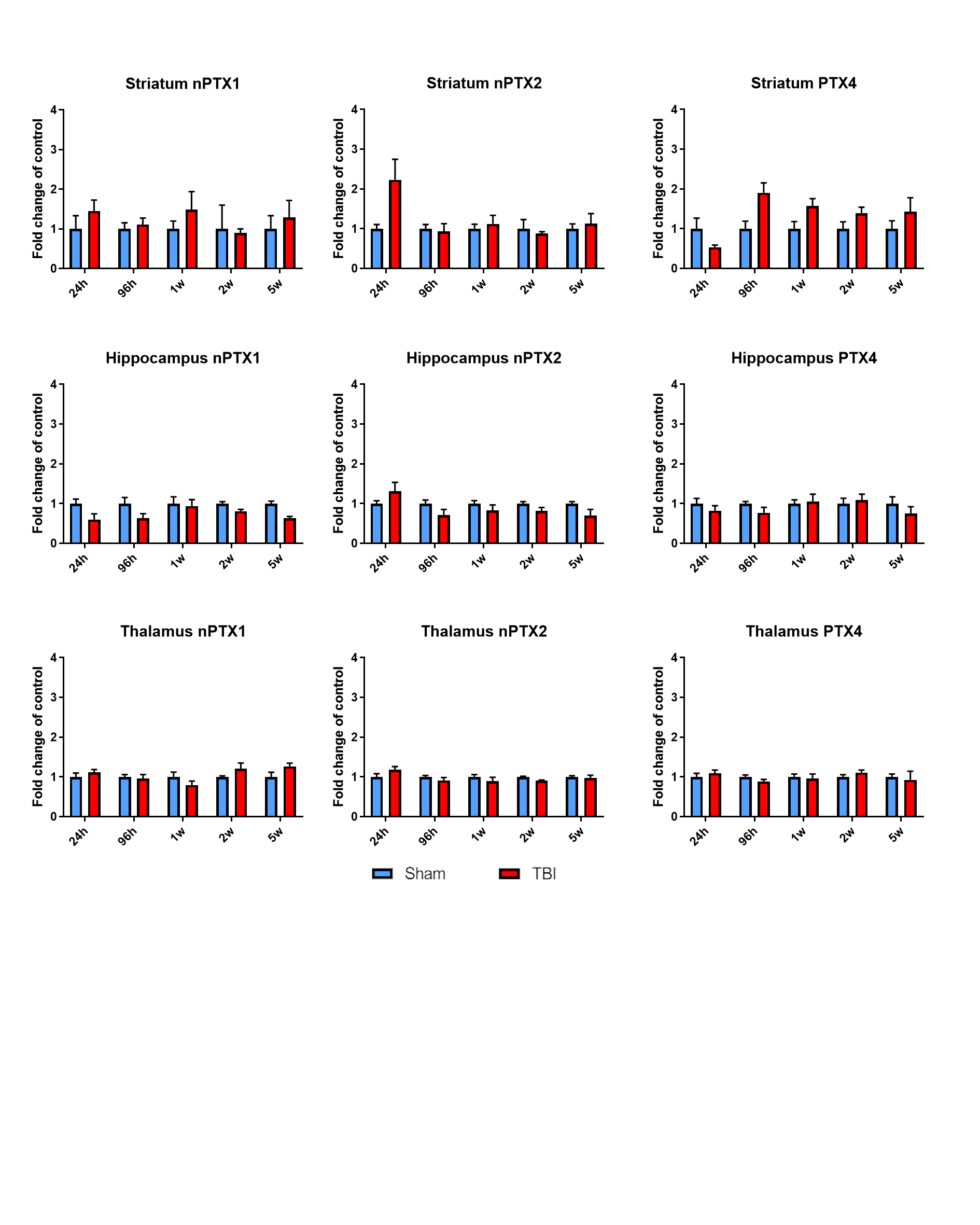


**Additional Figure 1. Long pentraxins nPTX1, nPTX2 and PTX4 mRNA expression in striatum, hippocampus and thalamus.** Data is presented as mean ± S.E.M, n = 6-8. For nPTX1 in striatum, nPTX1, nPTX2 and PTX4 in hippocampus, nPTX1 and nPTX2 in thalamus, computations assume that all rows are sampled from populations with the same scattered SD. Multiple t-test followed by Holm-Sidak post hoc test = ns.

| **CORTEX (sham and TBI group)** | **SAP** | **CRP** |
| --- | --- | --- |
| **24h, 96h, 1w, 2w, 5w after TBI** | **ND** | **ND** |

**Additional Table 1. Short pentraxins SAP and CRP mRNA expression in brain cortex.** SAP and CRP were unchanged in the il-cortex as measured in different time points (24h, 96h; 1w, 2w and 5w) after TBI compared to sham mice. ND – not detected.


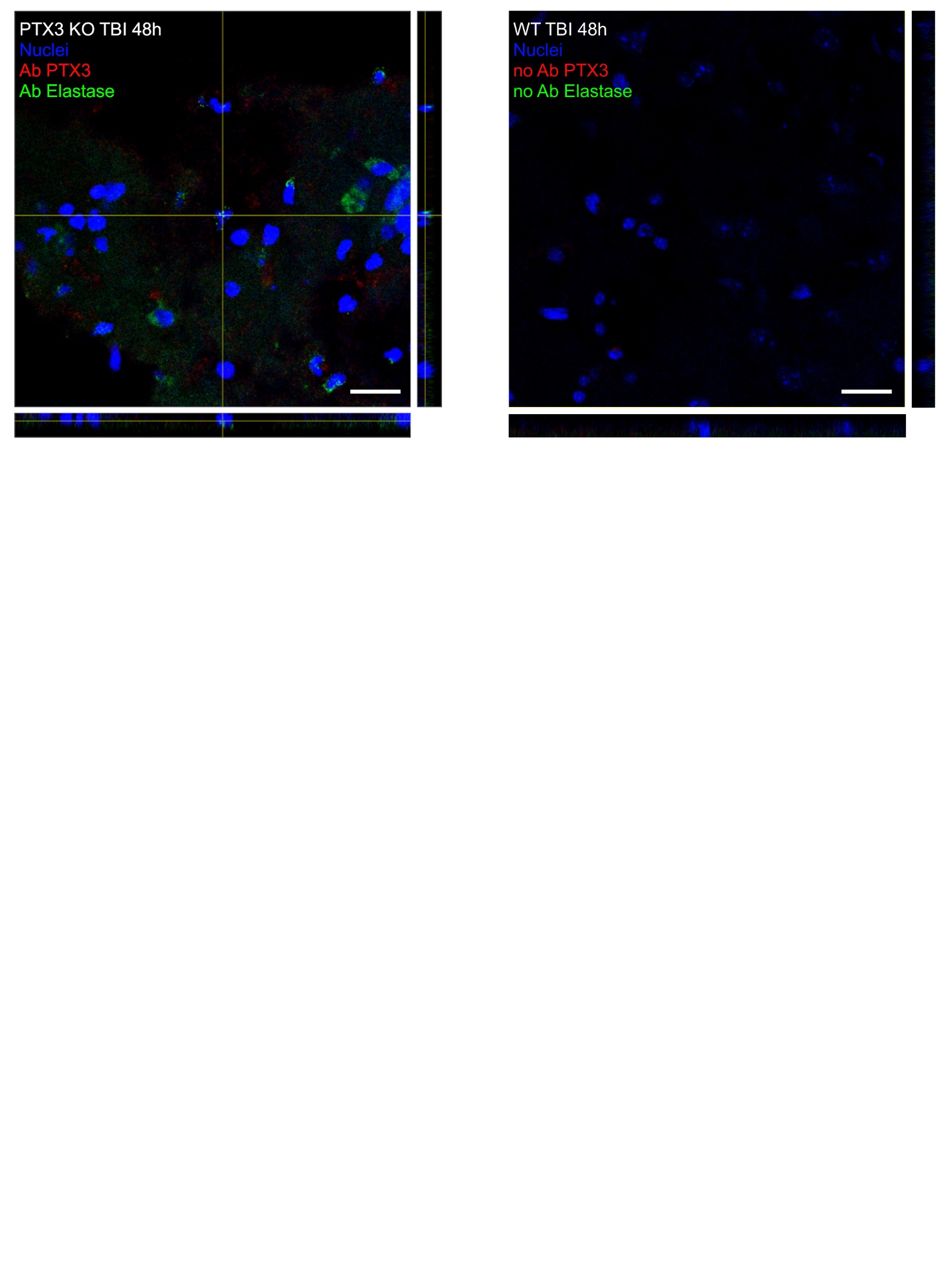


**Additional Figure 2: Immunofluorescence controls.** PTX3 showed no signal in PTX3 KO mice and in WT without primary antibody, confirming staining specificity. Elastase in PTX3 KO is in green, while no antibody was used in WT control. Nuclei are in blue. Images are representative of at least two independent experiments. Scale bar = 20 µm.


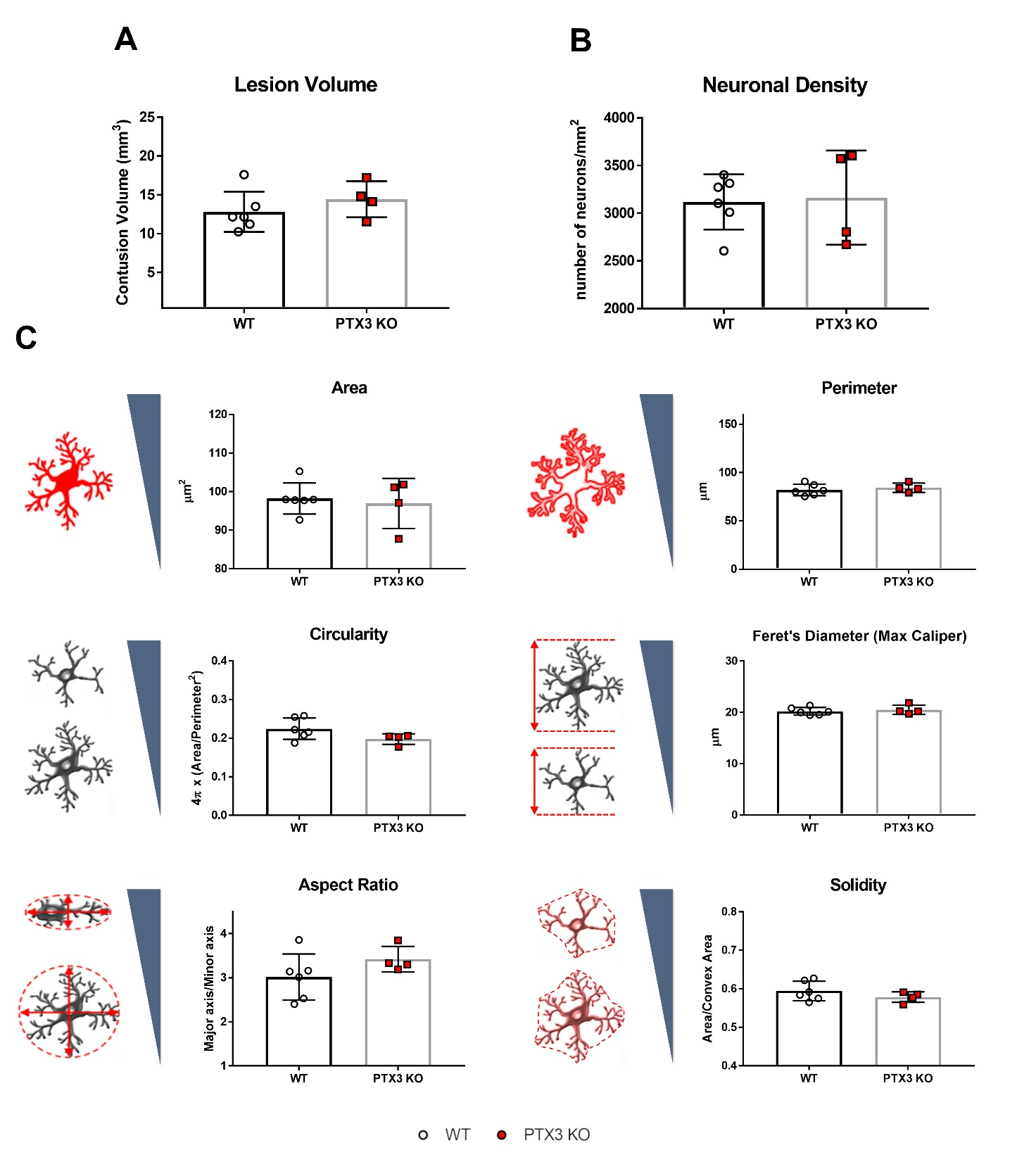


**Additional Figure 3: PTX3 depletion did not modify the outcome 1 week after TBI.** A) 1 week after TBI, PTX3 depletion did not affect the lesion volume compared to WT mice. Data is presented as mean ± SEM, n = 5 (TBI WT) n = 4 (TBI PTX3 KO). Unpaired t-test = ns. B) WT and PTX3 KO mice did not show difference in neuronal density (neurons per mm^2^) in the lesioned cortex 1 week after TBI. Data is presented as mean ± SEM, n = 5 (TBI WT) n = 4 (TBI PTX3 KO). Unpaired t-test = ns. C) Shape descriptors of CD11b positive cells did not show difference between WT and PTX3 KO mice 1 week after TBI. Data is presented as mean ± SEM, n = 5 (TBI WT) n = 4 (TBI PTX3 KO). Unpaired t-test = ns.
